# Sex-related communicative functions of voice spectral energy in human chorusing

**DOI:** 10.1098/rsbl.2023.0326

**Published:** 2023-11-08

**Authors:** Peter E. Keller, Jennifer Lee, Rasmus König, Giacomo Novembre

**Affiliations:** ^1^ Center for Music in the Brain, Department of Clinical Medicine, Aarhus University, Aarhus 8000, Denmark; ^2^ The MARCS Institute for Brain, Behaviour and Development, Western Sydney University, Penrith South, Australia; ^3^ Queensland Aphasia Research Centre, University of Queensland, Brisbane, Queensland, Australia; ^4^ Saxon State Ministry for Culture, Germany; ^5^ Neuroscience of Perception and Action Lab, Italian Institute of Technology, Rome, Italy

**Keywords:** music, vocal expression, human evolution, social cognition, non-verbal communication, singer's formant

## Abstract

Music is a human communicative art whose evolutionary origins may lie in capacities that support cooperation and/or competition. A mixed account favouring simultaneous cooperation and competition draws on analogous interactive displays produced by collectively signalling non-human animals (e.g. crickets and frogs). In these displays, rhythmically coordinated calls serve as a beacon whereby groups of males ‘cooperatively’ attract potential female mates, while the likelihood of each male competitively attracting an actual mate depends on the precedence of his signal. Human behaviour consistent with the mixed account was previously observed in a renowned boys choir, where the basses—the oldest boys with the deepest voices—boosted their acoustic prominence by increasing energy in a high-frequency band of the vocal spectrum when girls were in an otherwise male audience. The current study tested female and male sensitivity and preferences for this subtle vocal modulation in online listening tasks. Results indicate that while female and male listeners are similarly sensitive to enhanced high-spectral energy elicited by the presence of girls in the audience, only female listeners exhibit a reliable preference for it. Findings suggest that human chorusing is a flexible form of social communicative behaviour that allows simultaneous group cohesion and sexually motivated competition.

## 1. Introduction

Music, as a communicative medium for individual and collective expression, constitutes a microcosm of social interaction [1]. Evolutionary accounts propose that music fulfils social functions in group cohesion, coalition signalling, courtship, infant–caregiver bonding and cultural identity [2–6]. These functions rely on capacities supporting the synchronization of rhythms and harmonization of pitches among individuals singing or playing instruments together [7–11]. As in other social animals [12–14], such capacities were presumably selected for benefits related to cooperation, competition, or mixtures of these [15–19].

On the mixed account, music can function both cooperatively and competitively, and doing so simultaneously allows different forms of communication to occur in parallel at group and individual levels [20]. Taking a comparative approach [11,21,22], this hypothesis was motivated by observations that in some non-human animals (e.g. flashing fireflies and chorusing crickets and frogs), simultaneous cooperation and competition is manifest in rhythmically coordinated communal displays produced by groups of males to attract female mates [23–25].

In these displays, seemingly cooperative coordination, which increases the salience of the collective broadcast (beacon effect) [26,27], can be a side-effect of sexually motivated competition whereby individual males produce earlier or more intense signals that mask rival signals (precedence effect) [14] while triggering timing adjustments in neighbouring individuals [28,29]. The mechanisms that govern these adjustments affect the inter-individual phase alignment of signals, and predominantly give rise to synchrony or alternation [30].

Non-human chorusing may have evolved in response to multi-level selection pressures [31]. Rhythmically coordinated clusters of males who produce more attractive beacons than neighbouring clusters could be favoured by group selection [32]. Mechanisms that give rise to precedence effects in response to female preferences for energetic male signals could be favoured by individual selection [33–35]. Conspecific males may, nevertheless, be sensitive to precedence effects because this facilitates eavesdropping on others' courtship signals [36–39].

Support for the hypothesis that human music can function to support simultaneous cooperation and competition was found in a study with a renowned boys choir, the St Thomas Choir of Leipzig [20]. Results indicated that the basses—the oldest boys with the deepest voices—exhibited increased energy in the ‘singer's formant’ (2500–3500 Hz) frequency region of the vocal spectrum [40,41] when girls were included in an otherwise male audience. Because the singer's formant adds an attractive ringing quality to the voice [42–44], the observed enhancement could reflect an attempt by sexually mature boys to compete for female attention without undermining collaborative musical goals.

The current study addressed the functional relevance of this behavioural modification by testing whether the enhanced singer's formant is detectable by listeners, and whether preferences for it are affected by sex. In two online perceptual studies, female and male listeners (*N* = 2247) were presented with pairs of audio excerpts from the original choir performances with or without girls in the audience. Two musical pieces that varied in degree of rhythmic unison between voices (approximating synchrony versus alternation) were included to test the generality of effects. Listeners either reported which excerpt they believed was sung in the presence of girls (sensitivity study) or which excerpt they preferred (preference study). Female sensitivity and preference for the enhanced singer's formant would be consistent with beacon and precedence effects, while male sensitivity without preference would be consistent with eavesdropping.

## 2. Methods

### (a) Participants

The participants were 679 females (aged 12–71 years) and 481 males (aged 17–81) in the sensitivity study, and 655 females (aged 13–78) and 432 males (aged 12–86) in the preference study, including individuals with and without musical training (see electronic supplementary material [45]).

### (b) Design

The sensitivity study tested the ability of female and male listeners to identify which item from pairs of excerpts of choral performances of two musical pieces was sung in the presence of girls in the audience. The independent variables were listener sex (female or male) and musical piece (Chorale or Fugue), and the dependent variable was the percentage of excerpt pairs in which listeners selected the correct item (i.e. the excerpt sung with females present). The preference study assessed which items were preferred.

### (c) Materials

This stimulus set included 12 pairs of items consisting of audio excerpts from performances of a Chorale and a Fugue—sung by an elite boys choir to audiences in which girls were present or absent [20]. The pieces were composed by Johann Sebastian Bach for a four-part choir setting comprising soprano, alto, tenor, and bass voices. The Chorale's homophonic texture requires rhythmic unison and strict synchrony between parts, whereas the Fugue's polyphonic texture has greater rhythmic independence between parts. The singers were 16 members of the St Thomas Choir of Leipzig in Germany: four sopranos (aged 12–13 years); four altos (aged 12–16); four tenors (aged 16–18); four basses (aged 16–19). The performances were recorded with a video camera, from which audio was extracted.

**Figure 1.**
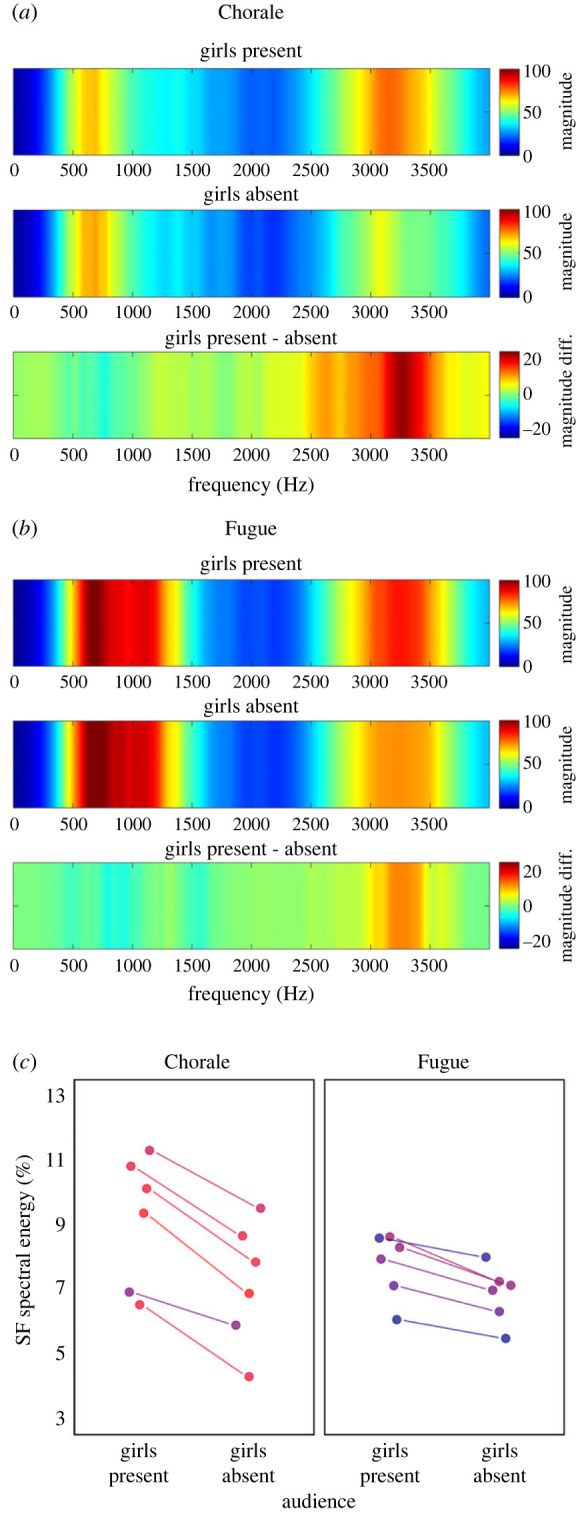
Audio analyses show increases in high-frequency spectral energy for male choir performances when girls were in the audience. (*a*) Time-averaged spectra (filtered using the Terhardt outer ear model [47] to reflect human hearing sensitivity) for audio recordings of the homophonic Chorale piece with rhythmic synchrony between vocal parts, showing increased energy in the 2500–3500 Hz singer's formant frequency region when girls were present versus absent. (*b*) Corresponding spectra for a polyphonic Fugue, with rhythmically independent vocal parts. (*c*) Percentage of energy in the singer's formant (SF) region for individual stimulus excerpts from the two pieces, showing less pronounced SF energy for the Fugue.

Brief excerpts (3–6 s duration) were selected from the full choir recordings and compiled into 12 stimulus pairs wherein one item came from a performance sung with girls present and the other with girls absent from the audience. Excerpts from performances sung with girls present occurred as the first item in half of the stimulus pairs and as the second item in the other half. Additional items from the Chorale were selected for a practice trial and to check the reliability of listener responses. Acoustic analyses of the 12 main items (see electronic supplementary material [45,46]) confirmed that energy in the singer's formant region was higher for excerpts sung with girls present, and additionally revealed that the effect was stronger for the Chorale than the Fugue (figure 1*a–c*), possibly because greater spectral change is required to stand out from the homogeneous than the polyphonic texture. Performance timing and overall intensity did not vary with the presence of girls (electronic supplementary material [45,46]).

### (d) Procedure

The sensitivity study and the preference study were conducted on an online survey platform that participants accessed remotely on their own devices. In the sensitivity study, participants were informed that they would be presented with pairs of short excerpts from two live concerts, that girls were present in the audience for only one concert, and that the task was to indicate which excerpt was more likely to come from that concert. For the preference study, participants were informed that they would be presented with pairs of excerpts from two live concerts, and that the task was to indicate which performance they preferred. The use of headphones was recommended.

For the test items that followed, participants were presented a text prompt, a media player, and two clickable response buttons (labelled ‘Performance 1’ and ‘Performance 2’). The 12 stimulus items were blocked by musical piece (Chorale and Fugue), with block order randomized across participants. Presentation order of the six pairs of excerpts (Chorale or Fugue) within each test block was also randomized (with the reliability check item interspersed). Following the listening task, a background questionnaire was presented to collect information about participant age, sex, musical experience (formal training and choir participation), and cultural background (European, non-European, or mixed).

## 3. Results

### (a) Sensitivity

**Figure 2.**
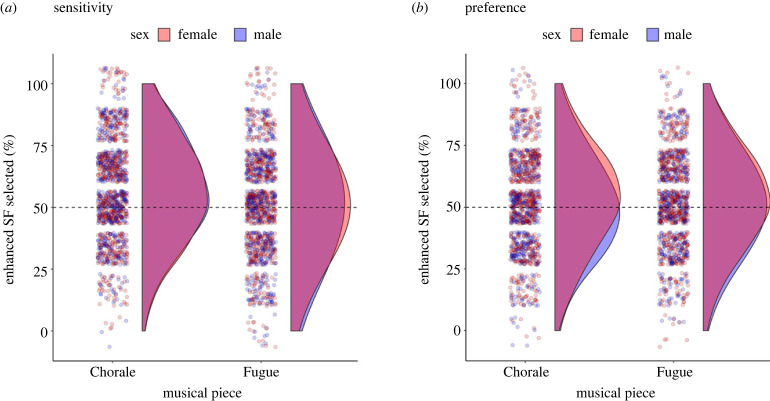
Female and male listeners were similarly sensitive to increased high-frequency spectral energy in the singer's formant (SF) region in male choir performances, but only female listeners preferred the enhancement. (*a*) Listener sensitivity scores, indicating that female and male listeners identified excerpts from performances with girls in the audience (and with enhanced SFs) at greater than chance level for the Chorale (i.e. the bulk of female and male density plots sits above 50%) but not the Fugue. (*b*) Listener preference scores, showing that excerpts with the enhanced SF were preferred at greater than chance level by female listeners (higher density above 50%), but not male listeners, for both pieces.

Listener sensitivity data, indicating the percentage of items where participants correctly selected the excerpt with an enhanced singer's formant (i.e. from performances sung with girls in the audience), are displayed in figure 2*a*. A Wilcoxon test on all data pooled (averaged across musical pieces) revealed that sensitivity scores were overall significantly higher than expected by chance (50%) (*V* = 277274, *p* < 0.001). Binomial generalized linear mixed model (GLMM) analyses of these data (see electronic supplementary material, [45,46]) indicated that models that included listener sex, musical piece, listener age, and musical experience (plus interactions) as fixed factors (Full Models 2 & 3 in electronic supplementary material, table S4), and participant and item as random effects, had greater explanatory power than alternative models. However, none of the fixed factors (or interactions) were significant predictors of sensitivity scores in these best-fitting models. Additional Wilcoxon tests revealed that sensitivity scores were significantly higher than chance for female listeners and male listeners for the Chorale (females: *V* = 79800, *p* < 0.001; males: *V* = 38815, *p* < 0.001), but not for the Fugue (females: *V* = 57710, *p* = 0.071; males: *V* = 32554, *p* = 0.418). Listeners were thus generally sensitive to the enhanced singer's formant for the homophonic Chorale, but results for the polyphonic Fugue were less reliable (possibly due to less pronounced energy modulation; figure 1*c*).

### (b) Preference

Listener preference data, indicating the percentage of items where the excerpt with an enhanced singer's formant was selected as being preferred, are displayed in figure 2*b*. Overall preference scores were statistically significantly higher than chance (*V* = 220124, *p* < 0.001). GLMM analyses (see electronic supplementary material, [45,46]) revealed that a model including listener sex and musical piece (and their interaction) as fixed factors (Full Model 1 in electronic supplementary material, table S7) had greater explanatory power than a reduced model containing only random effects (participant and item). Alternative models that additionally included listener age, musical experience, and cultural background as fixed factors did not increase explanatory power significantly. For the best-fitting model, there was a statistically significant effect of listener sex on preference scores (estimate = 0.133, *SE* = 0.051, *z* = 2.60, *p* = 0.009) but no further significant effects. Additional tests confirmed that female scores were significantly above chance for the Chorale (*V* = 59954, *p* < 0.001) and the Fugue (*V* = 57862, *p* < 0.001). Male scores were not significantly different from chance for either piece (Chorale: *V* = 21661, *p* = 0.928; Fugue: *V* = 23514, *p* = 0.190). Female listeners thus exhibited a preference for the enhanced singer's formant that generalized across musical pieces, whereas males did not show reliable preferences.

## 4. Discussion

Our results indicate that female and male listeners are sensitive to the enhanced singer's formant in male chorusing, but only females prefer it. Boosting high-frequency spectral energy may thus constitute an attempt by male singers to establish a privileged social communication channel with female listeners.

Overall findings—which generalize across listener age, musical experience, and cultural background—are consistent with characterizing human chorusing as a form of social behaviour that allows selfish competitive drives to be pursued without disrupting cooperative behaviour. In this multi-level display, sexual competition at the individual level coexists with social cooperation at the group level [20]. Our interpretation draws an analogy with chorusing displays by groups of males to attract female mates in other species [23–25] (though here via spectral rather than temporal or amplitude effects). Female listener sensitivity and preference for the enhanced singer's formant might correspond to female responsiveness to prominent male signals in these species [33]. Male listener sensitivity without preference may be akin to the ability of eavesdropping non-human males to detect others' courtship signals [37,39].

The observed differences for the homophonic Chorale and the polyphonic Fugue are reminiscent of distinct forms of non-human chorusing characterized by synchrony or alternation [32,48]. These two coordination modes are prominent in human music [49], where multi-part textural variations range from rhythmic unison (with voices singing different pitches in harmony or the same pitches, as in chanting) to complex interlocking rhythms [50]. Reliable female listener preferences despite reduced detectability with the polyphonic Fugue (with weaker singer's formant enhancement) suggest implicit processing consistent with perceptual biases in other species [28].

The current proposal that music can simultaneously fulfil cooperative and competitive functions supplements existing evolutionary accounts, which rely to differing degrees on cooperation or competition. These accounts appeal to different selection models (from sexual to multilevel) [2,6,7,18,51,52], with proposals favouring sexual selection of male courtship displays attracting criticism, partly because musicality is not sexually dimorphic [2,6,53]. Our focus on male chorusing might therefore appear controversial. Moreover, the enhanced singer's formant in our choir recordings was produced by members of the bass section [20], i.e. older boys with deep voices.

Basses possibly have relatively high levels of testosterone [54], which lengthens the male vocal tract by stimulating a secondary descent of the larynx during puberty [55,56]. The resulting lower fundamental frequency and reduced dispersion in formant frequencies [57] can increase perceived vocal attractiveness and dominance [58,59], perhaps by exaggerating body size [60–62], though this effect might be specific to speech and possibly reverses in singing [63]. Higher-voiced tenors may thus hold an advantage and, furthermore, might not require additional enhancement because the centre frequency of their singer's formant cluster is higher (hence more salient) than in basses [40].

Importantly, while enhancement of the singer's formant is typically associated with male voices, related spectral modulations can occur in females [64–66], consistent with sexual non-dimorphism [6]. It would therefore be prudent to study female and mixed-sex chorusing to test whether male listeners have preferences for corresponding modulations in female voices before proposing specific selection mechanisms. Potential effects of sexual orientation for both singers and listeners constitute another worthwhile topic for future research.

Evolutionary considerations aside, our findings demonstrate flexibility in human vocal expression for social communication, specifically in the modulation of spectral properties influencing voice timbre, which has received less attention than pitch and timing. These properties transmit personal information [67,68] and play a role in mate attraction [59,69]. Speakers spontaneously alter their voices when interacting with desirable conversation partners [70–73], and manipulate vocal fundamental frequency and formants to sound dominant, larger, and sexually appealing [60,74]. Related phenomena may occur in instrumental music, but the ancient and universal status of singing [75–77]—including prevalent male chorusing [78]—as well as its powerful modulatory effects on social behaviour [79–81], make it especially apt for studying communicative flexibility. Singers tailor their vocal qualities to their expressive intentions [40], and present results show that they can do so in a manner that facilitates parallel acoustic channels of social communication. Human chorusing thus simultaneously permits competitive and cooperative goals at individual and group levels, thereby providing a platform that supports complex social interaction through music.

## Data Availability

Stimulus files for individual audio excerpts, raw data from the sensitivity and preference online listening studies, and scripts for statistical analyses are available from the Dryad Digital Repository: https://doi.org/10.5061/dryad.jh9w0vthn [45]. Additional details for methods and results are provided in the electronic supplementary material [46].
